# Knowledge and HIV-Related Stigmatizing Attitudes Among Healthcare Workers: A Cross-Sectional Study

**DOI:** 10.3390/bs16091681

**Published:** 2026-09-18

**Authors:** Zulal Yavuz Akay, Fatma Ozlem Kandemir, Mustafa Serhat Sahinoglu, Tugce Simsek Bozok, Kasim Akay

**Affiliations:** 1 Department of Infectious Diseases and Clinical Microbiology, Osmaniye Training and Research Hospital, 80010 Osmaniye, Turkey; 2 Department of Infectious Diseases and Clinical Microbiology, Mersin University Faculty of Medicine, 33110 Mersin, Turkey; 3 Department of Obstetrics and Gynecology, Duzici State Hospital, 80600 Osmaniye, Turkey

**Keywords:** HIV/AIDS, healthcare workers, stigma, discrimination, knowledge, cross-sectional study

## Abstract

**Background:** Healthcare workers are central to the care of people living with HIV/AIDS (PLHIV), yet their own stigmatizing attitudes can undermine it. This cross-sectional study assessed HIV/AIDS knowledge and stigmatizing attitudes toward PLHIV among healthcare workers and the factors associated with stigma. **Methods:** All physicians and nurses employed at three public hospitals in Mersin, Turkey (*N* = 2230), were invited to participate; because participation was voluntary and anonymous, the analysed sample was self-selected and is best characterised as a convenience sample. Data were collected over 45 days using an anonymous, self-administered questionnaire hosted on an online survey platform, comprising a 10-item HIV/AIDS knowledge questionnaire adapted from a previously used instrument and the Health Care Provider HIV/AIDS Stigma Scale (HPASS; Turkish version), which measures stereotyping, discrimination, and prejudice. Associations were examined using group comparisons, correlations, and multivariable linear regression. **Results:** Among 460 participants (median age 35.0 years, IQR 31.0–44.0; 63.5% female; response rate 21.7%), knowledge assessment scores were not associated with any stigma subdimension; instead, older age, being married, and greater professional experience were associated with higher stereotyping and discrimination, with age and experience remaining significant after multivariable adjustment (*p* < 0.05). Notably, 81.1% felt they would need to inform other staff when encountering a PLHIV, which may raise confidentiality concerns; 67.4% believed HIV is mostly acquired through risky behaviours, and only 69.1% were comfortable working with an HIV-positive colleague. **Conclusions:** Stigmatizing attitudes were common and were associated with age and professional experience rather than with knowledge assessment scores. These findings generate the hypothesis that interventions addressing the attitudinal and professional-culture dimensions of stigma—particularly among more senior staff—may be more effective than information provision alone. Because the design was cross-sectional and the knowledge questionnaire had limited validity evidence, longitudinal and interventional studies are needed to test this hypothesis.

## 1. Introduction

Human Immunodeficiency Virus (HIV) has continued to be one of the most significant global public health issues since its first identification in 1981. Currently, approximately 40.8 million people are estimated to be living with HIV worldwide (World Health Organization, 2025). Despite significant medical and technological advances in the HIV pandemic, people living with HIV (PLHIV) still face social stigma, discrimination, and consequent serious psychosocial challenges (Earnshaw & Chaudoir, 2009).

Stigmatization and discrimination, identified by the World Health Organization (WHO) as priority intervention areas, constitute critical barriers to controlling the HIV epidemic (World Health Organization, 2016). This situation not only negatively affects individual health outcomes but also significantly limits the effectiveness of community-level HIV prevention, testing, and treatment strategies (Mahajan et al., 2008).

While stigma and discrimination related to HIV/AIDS vary across cultures and societies, they are observed in virtually every community (Zorlu Günok & Çalım, 2012). According to recent UNAIDS (Joint United Nations Programme on HIV and AIDS) survey data from 42 countries, on average, nearly half (47%) of people exhibit discriminatory attitudes toward PLHIV (Joint United Nations Programme on HIV/AIDS, 2024). WHO and UNAIDS have set targets for 2030 of ‘zero new HIV cases’, ‘zero discrimination’, and ‘zero AIDS-related deaths’. Furthermore, the report emphasizes that inequalities underlying stigmatization, discrimination, and HIV-related criminalization increase the likelihood of PLHIV dying from AIDS-related diseases and make it difficult to control the epidemic. Projections suggest that if progress is made at the societal and individual levels toward stigma and discrimination globally, a reduction of 1.7 million AIDS-related deaths and 2.5 million new HIV infection cases is expected by 2030 (Joint United Nations Programme on HIV/AIDS, 2021).

The healthcare sector is one of the primary settings in which PLHIV experience stigma and discrimination (Cianelli et al., 2011), and such experiences adversely affect their treatment continuation and follow-up, social life, and mental health (Ataç & Buzlu, 2017; Zhao et al., 2019). Conceptually, HIV-related stigma is not a single construct but a multidimensional phenomenon with cognitive, emotional, and behavioural components, commonly operationalised as stereotyping (negative beliefs about PLHIV), prejudice (negative feelings toward them), and discrimination (negative actions or intentions) (Wagner et al., 2014). These components arise through partly distinct pathways: cognitive stereotyping is often fuelled by knowledge deficits, particularly misconceptions about transmission routes and prevention; prejudice is driven more by fear of occupational infection—and, to a lesser extent, by moral judgements that associate HIV with socially disapproved behaviour (Stutterheim et al., 2014); and discrimination reflects the translation of these beliefs and feelings into action or intended action (Earnshaw & Chaudoir, 2009). This distinction is important because it implies that improving factual knowledge may reduce some, but not necessarily all, dimensions of stigma, an issue directly examined in the present study. It should be emphasised at the outset that the instrument used here captures self-reported attitudes and behavioural intentions rather than directly observed clinical conduct. Throughout this paper, “discrimination” therefore refers to self-reported discriminatory intentions as measured by the HPASS, and not to discriminatory behaviour observed in practice; this distinction is maintained in the interpretation of all findings.

Fear of HIV-related stigmatization during the testing and diagnosis phase significantly reduces testing rates among at-risk individuals (Logie et al., 2017). Research shows that fear of stigmatization reduces HIV testing by 25–45%, resulting in late diagnosis and increased morbidity–mortality rates (Hatzenbuehler et al., 2011). Regarding treatment initiation and adherence, stigmatization concerns negatively affect PLHIV’s decisions to start and continue antiretroviral therapy (ART) (Katz et al., 2013).

Turkey is a low-HIV-prevalence country, with an estimated adult prevalence below 0.1%. However, the epidemiological trajectory is upward: according to the Turkish Ministry of Health, over 80% of the 45,835 HIV notifications recorded between 1985 and November 2024 occurred in the past decade, and new diagnoses have continued to rise (Yaylali & Erdogan, 2025). This growing case load makes the attitudes of healthcare workers toward PLHIV increasingly relevant in the Turkish context.

HIV-related stigma among healthcare workers remains a widespread problem globally, and in Turkey studies addressing it are still relatively few (Ataç & Buzlu, 2017; Hekimoğlu et al., 2017; Özçelik et al., 2025). The most comprehensive Turkish study to date, conducted by Koseoglu Ornek et al. (2020) in Istanbul among 405 healthcare workers, found that fear-driven stigma was significantly associated with age, occupation, and work experience. However, most existing studies report a single overall stigma score and pay limited attention to how its distinct components vary across professional groups, or to whether greater knowledge is genuinely accompanied by less stigma (Bozkurt & Bayırlı Turan, 2020; Gökengin et al., 2017; Köseoğlu Örnek & İşcan Kocamış, 2022; Yaman & Güngör, 2013).

The present study was prompted by a specific clinical observation—reluctance among surgical staff to provide operative care to a person living with HIV—which raised the question of whether stigmatizing attitudes differ between physicians and nurses, between medical and surgical branches, and across levels of seniority. Accordingly, we aimed to assess HIV/AIDS knowledge and stigmatizing attitudes (stereotyping, discrimination, and prejudice) among physicians and nurses, to examine their sociodemographic and occupational correlates using multivariable analysis, and to determine whether higher knowledge assessment scores are associated with lower stigma.

## 2. Materials and Methods

### 2.1. Study Design

This was a cross-sectional, descriptive survey study conducted between 15 August and 30 September 2024 among physicians and nurses employed at three public hospitals in Mersin, Turkey. The study was designed and reported in accordance with the Strengthening the Reporting of Observational Studies in Epidemiology (STROBE) guideline for cross-sectional studies; the completed STROBE checklist is provided as Supplementary Materials (Table S1).

### 2.2. Setting

The study was carried out in Mersin, a metropolitan city on the Mediterranean coast of southern Turkey with a population of approximately 1.9 million that serves as a regional healthcare hub for the surrounding provinces. Three public hospitals were included: Mersin University Hospital, Mersin City Training and Research Hospital, and Toros State Hospital. These institutions were selected purposively because they represent the three principal tiers of the public healthcare system in the province: Mersin University Hospital is the only university hospital in the city, Mersin City Training and Research Hospital is its only training and research hospital, and Toros State Hospital is its largest central state hospital. Together they employ the majority of the physicians and nurses working in the city centre, and their inclusion was therefore intended to capture a broad range of healthcare worker profiles rather than to sample from a larger pool of comparable institutions. In all three hospitals, HIV-positive patients are followed up by the Department of Infectious Diseases and Clinical Microbiology; no dedicated HIV outpatient clinic exists at any site, and PLHIV are managed within the general infectious disease outpatient setting alongside other patient groups.

### 2.3. Participants and Sampling

Eligible participants were physicians and nurses actively employed at one of the three participating hospitals during the study period. Other healthcare occupational groups (e.g., pharmacists, laboratory technicians, and administrative staff) were not eligible, and no minimum duration of employment was required. Incomplete submissions were excluded, and only forms completed in full were retained for analysis.

All eligible physicians and nurses at the three hospitals (*N* = 2230) were invited to participate, corresponding to a total-population (census) invitation. Because participation was voluntary and anonymous, the analysed sample was ultimately self-selected and is therefore best characterised as a convenience sample. The minimum required sample size was estimated using Cochran’s formula for a single proportion, *n*_0_ = *Z*^2^·*p*·(1 − *p*)/*e*^2^, with *Z* = 1.96 (95% confidence level), *e* = 0.05 (margin of error), and *p* = 0.50 (the value that maximises the required sample size when no prior estimate is available). This yielded n_0_ = 384, which was adjusted for the finite source population using the finite population correction, *n* = *n*_0_/[1 + (*n*_0_ − 1)/*N*], giving a corrected minimum of approximately 328 participants. The 460 fully completed forms obtained exceeded this requirement.

### 2.4. Data Collection

Data were collected using an anonymous, self-administered electronic questionnaire hosted on an online survey platform (Google Forms). The survey link was distributed to all eligible physicians and nurses at the three participating hospitals by email and through professional communication networks, and the form remained open for 45 days. To prevent duplicate submissions, the platform was configured to accept only one response per participant.

### 2.5. Measures

The data collection form consisted of three parts.

**Sociodemographic and professional characteristics.**

This section recorded age, gender, marital status, profession (physician or nurse), physician title where applicable (faculty member, specialist physician, or research assistant), institution of employment, and department. Departments were classified as internal (medical) or surgical branches, the latter comprising specialties that routinely perform invasive or operative procedures. Professional experience was recorded as the total number of years worked in the profession. Care experience with PLHIV was defined as having personally provided clinical care to at least one person living with HIV/AIDS at any point during professional practice, recorded as a yes/no item. Perceived HIV/AIDS knowledge was assessed with a single self-report item on which participants rated their own knowledge as “adequate”, “moderate”, or “inadequate”; this subjective self-assessment is conceptually distinct from the total score derived from the 10-item knowledge questionnaire.

**Knowledge questionnaire.**

HIV/AIDS knowledge was assessed using a 10-item questionnaire adapted from Vorasane et al. (2017), who employed a 10-item HIV/AIDS knowledge instrument that had previously been used among healthcare professionals. The items were translated into Turkish and adapted to the local context by the research team; a formal forward–backward translation procedure was not undertaken, and this is acknowledged as a limitation. Prior to administration, the adapted items were reviewed by an expert panel comprising an internal medicine specialist with 10 years of clinical experience, including the care of PLHIV, a general surgeon with 12 years of experience, and a nurse with 17 years of experience. Panel members assessed the clarity, comprehensibility, and contextual relevance of each item on the basis of their clinical experience; none had formal training in psychometrics or questionnaire development. A surgeon was deliberately included in this panel because the study was prompted by clinical concerns regarding willingness to provide surgical care to PLHIV, and because operative specialties carry a higher risk of intraoperative blood-borne exposure and may therefore hold distinct risk perceptions. Each correct answer was scored as 1 point and each incorrect or “I don’t know” answer as 0 points, yielding a total knowledge score ranging from 0 to 10. No quantitative content validity index (CVI/CVR) was calculated, no pilot testing was conducted, and no factor analysis was undertaken. The instrument therefore carries limited validity evidence in this sample, and the resulting total score is referred to throughout as a “knowledge assessment score” rather than as a validated measure of knowledge.

**HIV/AIDS stigma scale (HPASS).**

The Health Care Provider HIV/AIDS Stigma Scale (HPASS) is a 30-item Likert-type scale developed by Wagner et al. (2014) with a Turkish validity–reliability study conducted by Ceylan (2022). It consists of three subdimensions (stereotyping, discrimination, prejudice) and is evaluated on a 1–5 scale, with higher scores indicating higher levels of stigmatization. In accordance with the factor structure reported in the Turkish validation study (Ceylan, 2022), the stereotyping subdimension comprises 14 items (items 4, 8, 10, 12, 16, 18, 19, 22, 23, 24, 26, 27, 29, and 30; possible range 14–70), the discrimination subdimension comprises 12 items (items 3, 5, 6, 7, 11, 13, 14, 15, 17, 20, 25, and 28; possible range 12–60), and the prejudice subdimension comprises 4 items (items 1, 2, 9, and 21; possible range 4–20). Subdimension scores were calculated as the sum of the corresponding item scores. Item 15 (“I can work comfortably with a healthcare professional who has HIV”) is the only positively worded item of the scale and was reverse-coded prior to scoring, so that higher scores consistently indicate higher stigmatization across all items. Internal consistency of the HPASS was assessed using Cronbach’s alpha coefficient, calculated separately for the overall scale and each subdimension using IBM SPSS Statistics version 26 (IBM Corp., Armonk, NY, USA). The Cronbach’s alpha coefficient was reported as 0.915 in the original Turkish version. In this study, Cronbach’s alpha coefficients were calculated as 0.925 overall, and 0.856 for stereotyping, 0.880 for discrimination, and 0.710 for the prejudice subdimension.

### 2.6. Statistical Analysis

Data were analysed after transfer to digital format. The normality of continuous variables was assessed using the Shapiro–Wilk test. Because the data were not normally distributed, continuous variables are presented as median and interquartile range (IQR). For the Likert-type stigma items, median and IQR were reported for each item rather than percentage distributions. Categorical variables are expressed as frequencies and percentages.

Group comparisons of continuous variables were performed using the Mann–Whitney U test for two independent groups and the Kruskal–Wallis test for three or more independent groups. Where the Kruskal–Wallis test was significant, pairwise post-hoc comparisons were conducted using Dunn’s test with Bonferroni adjustment for multiple comparisons. Effect sizes were reported alongside the corresponding tests: the rank-biserial correlation (r) for Mann–Whitney U tests and epsilon-squared (ε^2^) for Kruskal–Wallis tests (Table S2). Correlations between variables were assessed using Spearman’s rank correlation coefficient. A two-sided *p*-value below 0.05 was considered statistically significant. All analyses were performed using IBM SPSS Statistics version 26.

Multivariable linear regression models were constructed separately for the stereotyping, discrimination, and prejudice subdimension scores, adjusting for age, sex, marital status, profession, work experience, institution, department, previous care experience with PLHIV, perceived knowledge, and knowledge assessment score. Collinearity was assessed using variance inflation factors. Because age and work experience were highly collinear, they were entered into separate models (Model 1: age; Model 2: work experience) rather than simultaneously. As the subdimension scores were not normally distributed, confidence intervals and *p* values for the regression coefficients were obtained using bias-corrected and accelerated (BCa) bootstrapping with 1000 resamples. Unstandardized regression coefficients (B) with 95% confidence intervals and model R^2^ values are reported.

### 2.7. Ethical Considerations

The study was conducted after obtaining approval from Mersin University Faculty of Medicine Clinical Research Ethics Committee with decision number 2024/422 dated 8 May 2024.

Additionally, necessary institutional permissions were obtained from the local health authority. Participants were informed about the purpose of the study, all data were collected anonymously and were used solely for scientific research purposes. Participation was voluntary. Written informed consent was not obtained due to the anonymous nature of the online survey; instead, an electronic informed consent statement was provided at the beginning of the questionnaire, and participants indicated their agreement to participate before proceeding. This consent procedure was approved by the Mersin University Faculty of Medicine Clinical Research Ethics Committee.

## 3. Results

All 2230 physicians and nurses employed at the three participating hospitals were invited to take part. A total of 483 questionnaires were submitted, of which 23 were excluded because they were incomplete. The final analysis therefore included 460 fully completed questionnaires (response rate 483/2230 = 21.7%; analysed proportion 460/2230 = 20.6%). Descriptive statistics regarding the sociodemographic and occupational characteristics of participants are presented in Table 1. Because the survey was anonymous and voluntary, sociodemographic information on non-responders was not available and responders and non-responders could not be compared directly; the potential for self-selection (non-response) bias is considered in the Limitations.

The study group was relatively young (median age 35.0 years) and predominantly female (63.5%); full sociodemographic and occupational characteristics are presented in Table 1. Notably, previous care for PLHIV was reported by 62.4% of participants overall but varied markedly by title, being highest among specialist physicians (70.7%) and lowest among faculty members (41.7%). Most participants rated their own HIV/AIDS knowledge as moderate (62.0%). The 37.6% of participants who had never provided care to PLHIV were distributed relatively evenly across professions (nurses 38.8%, physicians 36.4%) and departments (internal medicine 40.6%, surgical 33.8%).

The results of the 10-question survey measuring participants’ knowledge level about HIV/AIDS are summarized in Table 2. 90.2% of participants correctly stated that the infection can be transmitted through birth, 80.9% that condom use can prevent transmission, and 79.6% that those with other sexually transmitted diseases have a higher likelihood of being infected with HIV. Conversely, the items with the lowest correct response rates were related to the treatability of AIDS (52.2%), the preventability of transmission through treatment (61.5%), transmission via mosquito bites (59.1%), transmission through shared utensils such as cutlery and towels (63.7%), and transmission through breastfeeding (65.7%). These findings indicate persistent misconceptions regarding both HIV transmission routes and current treatment options among healthcare workers.

The median scores and interquartile ranges for items in the three subdimensions of the Health Care Provider HIV/AIDS Stigma Scale—stereotyping, discrimination, and prejudice—are presented in Table 3.

Table 3 presents median scores and interquartile ranges for each scale item on a 5-point Likert scale (1 = Strongly Disagree, 5 = Strongly Agree). To complement these central tendency measures, the proportion of participants who responded “Agree” or “Strongly Agree” (scores 4–5) is additionally reported for each item. Selected items of particular clinical relevance are highlighted below and should be interpreted alongside their corresponding median scores.

**Stereotyping subdimension:** 63.3% of participants stated that PLHIV should accept responsibility for becoming infected.

**Discrimination subdimension:** 81.1% of participants expressed that they would feel the need to inform other healthcare workers when encountering a PLHIV.

**Prejudice subdimension:** 67.4% of participants stated they believed HIV infection is mostly acquired through risky behaviors, and 75% indicated they thought individuals would not become infected with HIV if they displayed responsible behavior.

Percentages reported represent the proportion of participants who responded “agree” or “strongly agree” (scores 4–5) to selected items, while Table 3 presents median scores for all items.

Additionally, 69.1% responded “agree” or “strongly agree” to the statement that they could work comfortably with a healthcare professional living with HIV; because this is the only positively worded item of the scale, agreement here indicates the absence rather than the presence of stigma. Endorsement was operationally defined as a response of “agree” or “strongly agree” (a score of 4 or 5) on the recoded scale, that is, after item 15 had been reverse-coded; for that item, endorsement therefore corresponds to disagreeing with the statement, which applied to 11.1% of participants. On this definition, 97.8% of participants endorsed at least one of the 30 items, 96.7% at least two, 90.2% at least five, and 64.3% at least ten. Excluding item 15 altogether left these four proportions unchanged, indicating that the positively worded item did not contribute to the reported endorsement levels. These proportions are descriptive summaries of item endorsement and should not be interpreted as prevalence estimates of stigma, because the HPASS has not been validated as a diagnostic instrument and no clinical cut-off has been established.

The effects of participants’ sociodemographic and occupational characteristics on knowledge and stigmatization levels are presented in Table 4.

**Gender:** No significant difference was found between male and female participants in terms of knowledge level and stigmatization scores (*p* > 0.05).

**Marital status:** Married participants had significantly higher stereotyping, discrimination, and prejudice scores compared to single participants (*p* < 0.001, *p* < 0.001, *p* = 0.014, respectively).

**Institution:** The knowledge level of those working at university hospitals was found to be higher than those working at other hospitals (*p* < 0.001).

**Specialty:** The knowledge level of those working in internal medicine specialties was significantly higher than those in surgical specialties (*p* = 0.012).

**Care experience:** Those with care experience with PLHIV had higher knowledge levels than those without (*p* < 0.001).

**Occupational groups:** Physicians’ knowledge level was significantly higher than nurses (*p* < 0.001). However, nurses’ stereotyping and discrimination scores were found to be higher than physicians (*p* < 0.001 and *p* < 0.001, respectively). No significant difference was observed between nurses and physicians in prejudice scores (*p* = 0.343).

**Physician titles:** Although faculty members had the highest median knowledge score, this difference was not statistically significant among physician title groups (*p* = 0.277). Nevertheless, their stereotyping and discrimination scores were significantly higher than other physician groups (*p* < 0.001 for both). Post-hoc pairwise comparisons revealed that specialist physicians had significantly higher scores than research assistants in both stereotyping (*p* < 0.001) and discrimination (*p* < 0.001), and that faculty members had significantly higher scores than both specialist physicians and research assistants in these two subdimensions (all adjusted *p* < 0.01; Faculty vs. Specialist, stereotyping *p* = 0.001 and discrimination *p* < 0.001; Faculty vs. Research Assistant, both *p* < 0.001). In contrast, prejudice scores did not differ significantly among physician title groups (*p* = 0.382).

Effect sizes for all group comparisons are provided in Table S2. These were generally small; the two largest were the physician–nurse difference in knowledge assessment scores (r = 0.49, medium-to-large) and the differences in stereotyping and discrimination across physician titles (ε^2^ = 0.24 and 0.29, respectively; large). The associations of marital status and profession with the stigma subdimensions were small-to-medium (r = 0.15–0.30).

Correlation analyses among the study’s main variables are shown in Table 5. Spearman’s correlation analyses revealed statistically significant, strong positive correlations between age and stereotyping (rs = 0.767, *p* < 0.001) and discrimination (rs = 0.758, *p* < 0.001), and a weak positive correlation between age and prejudice (rs = 0.272, *p* < 0.001). Similarly, professional experience showed strong positive correlations with stereotyping (rs = 0.806, *p* < 0.001) and discrimination (rs = 0.787, *p* < 0.001), and a weak positive correlation with prejudice (rs = 0.271, *p* < 0.001). In contrast, no statistically significant correlation was found between knowledge score and any of the stigmatization subdimensions (*p* > 0.05).

Because age and work experience were strongly collinear (rs = 0.946; variance inflation factor > 16 when entered simultaneously), two parallel sets of multivariable linear regression models were constructed: Model 1 included age and Model 2 included work experience, with all other covariates identical. In both sets, the maximum variance inflation factor was below 2.0. Confidence intervals and *p* values were obtained using bias-corrected and accelerated (BCa) bootstrapping with 1000 resamples. The results are presented in Table 6.

After adjustment for sex, marital status, profession, institution, department, previous care experience with PLHIV, perceived knowledge, and knowledge assessment score, age remained independently associated with all three subdimensions (stereotyping *B* = 0.74, 95% CI 0.67 to 0.82; discrimination *B* = 0.70, 95% CI 0.64 to 0.76; prejudice *B* = 0.11, 95% CI 0.07 to 0.14; *p* = 0.001 for all). Work experience showed a comparable pattern in Model 2 (stereotyping *B* = 0.74, 95% CI 0.68 to 0.81; discrimination *B* = 0.68, 95% CI 0.62 to 0.74; prejudice *B* = 0.10, 95% CI 0.07 to 0.13; *p* = 0.001 for all).

In contrast, the occupational differences observed in the bivariate analyses were attenuated after adjustment, but not to the same extent in the two models. Faculty members did not differ significantly from nurses in either model (stereotyping Model 1: *B* = −2.65, *p* = 0.076; Model 2: *B* = −1.28, *p* = 0.384; discrimination Model 1: *B* = −2.25, *p* = 0.106; Model 2: *B* = −0.74, *p* = 0.605). After adjustment for age (Model 1), however, specialist physicians and residents still had significantly lower stereotyping and discrimination scores than nurses (specialist: *B* = −2.30, *p* = 0.008 and *B* = −1.95, *p* = 0.030; residents: *B* = −2.76, *p* = 0.004 and *B* = −3.48, *p* = 0.001, respectively), whereas after adjustment for work experience (Model 2) none of the occupational groups differed significantly from nurses (all *p* > 0.05). This pattern indicates that the higher stereotyping and discrimination scores among nurses were accounted for more fully by differences in work experience than by age.

The knowledge assessment score was not significantly associated with any of the subdimensions in any of the six models (*p* = 0.089–0.958), consistent with the bivariate findings. The models explained a substantial proportion of the variance in stereotyping (R^2^ = 0.596 and 0.630 for Models 1 and 2, respectively) and discrimination (R^2^ = 0.597 and 0.609), but only a small proportion of the variance in prejudice (R^2^ = 0.115 and 0.117).

## 4. Discussion

This study reveals the strong relationship between HIV/AIDS-related stigmatization and sociodemographic and occupational factors. Our findings indicate that stigmatizing attitudes were associated not solely with knowledge gaps but with age, marital status, and professional experience; in multivariable analysis, seniority—captured by age in one model and by professional experience in the other—was the correlate most consistently associated with stigma.

In this sample, endorsement of at least one HPASS item was near-universal (97.8%); as noted in the Results, however, this descriptive figure should not be read as a prevalence estimate of stigma. The more informative finding is that stereotyping, discrimination, and prejudice scores increased significantly with age and professional experience. Strong positive correlations were found between both variables and the stereotyping and discrimination subscales, whereas their correlations with prejudice were weak.

This finding contradicts the existing literature (Cholewik et al., 2026; Dong et al., 2018; Steinhaus et al., 2024; Yigit et al., 2025) and may be interpreted from different perspectives. Traditional attitudes and social prejudices regarding HIV/AIDS appeared more pronounced among older participants and those with longer professional experience, and this gradient persisted after multivariable adjustment (Table 6), indicating that it is not merely a by-product of other sociodemographic differences. Because most participants were too young to have practised during the early HIV era—the median age of the sample was 35 years—this pattern is unlikely to reflect direct personal exposure to the panic that surrounded the disease’s emergence in the 1980s. A more plausible interpretation is that fear-laden norms and risk perceptions formed in that period have been transmitted intergenerationally through clinical training, mentorship, and institutional memory; this remains a hypothesis requiring dedicated study.

When evaluated in terms of marital status, most studies in the literature have not found a statistically significant relationship between marital status and stigmatization (Kingori et al., 2017; Nice et al., 2024; Rongkavilit et al., 2010). A smaller number of studies have reported higher stigma among married participants (Harapan et al., 2013). In our sample, married healthcare workers likewise had higher stereotyping scores in unadjusted comparisons. This association was borderline, however, and not robust. It did not persist in Model 2. The bootstrap confidence interval also marginally included zero (see footnote to Table 6). Marital status is furthermore difficult to separate from seniority. Married participants were on average older and more experienced. Sociocultural factors may well contribute, but none were measured here. Identifying such mechanisms would require instruments designed for that purpose.

In our study, physicians’ knowledge assessment scores were significantly higher than those of nurses; this comparison, however, rests on a knowledge questionnaire adapted from a previously used instrument, with limited validity evidence in the present sample (see Limitations) and should therefore be regarded as preliminary. Nurses nonetheless had higher stereotyping and discrimination scores than physicians, consistent with previous reports (Bayrak et al., 2014; Rogers et al., 2014), whereas prejudice scores did not differ significantly between the two groups. A possible explanation is that nurses generally have more prolonged patient contact and perform invasive procedures more frequently than physicians, which may heighten their perceived risk of occupational transmission. In line with this, nurses are repeatedly identified as the occupational group with the highest rate of sharps injuries in healthcare settings (Ergül, 2018), a factor that may reinforce risk perception and, in turn, stereotyping and discrimination. Such a mechanism is unlikely to operate in isolation: more limited HIV/AIDS content in nursing curricula relative to medical training, less in-service education, lower professional autonomy, and reduced participation in clinical decision-making may also contribute. These occupational comparisons should nevertheless be interpreted cautiously, since the groups differ substantially in age and work experience. These two variables were so highly correlated (rs = 0.946) that they could not be entered into the same model and were examined in alternative models; they are therefore best regarded as indicators of a single seniority dimension, and their respective contributions cannot be disentangled in this design.

In the comparison among physician titles, faculty members combined the highest knowledge assessment scores with the highest stereotyping and discrimination scores, and a seniority gradient was also apparent among physicians, with specialist physicians scoring higher than residents; prejudice scores, by contrast, did not differ significantly across titles (*p* = 0.382). This pattern makes the knowledge–behavior dissociation particularly visible at the most senior academic level: greater factual command of HIV did not translate into less discriminatory behavioural intent, running counter to reports of lower stigma among more senior personnel (Doka et al., 2017; Xie et al., 2019). Such a disconnect aligns with evidence that stigmatizing responses are driven more by implicit bias, moral–emotional appraisal, and long-established professional socialization than by cognitive knowledge alone (Pettigrew & Tropp, 2008), and its confinement to the behavioral and discriminatory domains—rather than to prejudicial beliefs—suggests that it reflects self-reported behavioural intent more than explicitly held attitudes. These observations must, however, be interpreted cautiously. Faculty members were few (*n* = 24) and differed systematically from junior physicians in age, seniority, work experience, and proximity to bedside care, reporting the lowest rate of previous care for PLHIV (41.7%). Critically, once age and work experience were accounted for in the multivariable models, the elevated scores among faculty members were no longer statistically significant (Table 6), indicating that the apparent title effect is largely attributable to seniority rather than to academic role itself. Whether academic position contributes independently of the seniority it typically accompanies remains a hypothesis to be tested in larger, purpose-designed studies.

Crucially, no statistically significant relationship was found between the knowledge assessment score and the stereotyping, discrimination, or prejudice subdimensions. This is one of the most important findings of the study, but it must be interpreted cautiously: the study did not evaluate any educational intervention, and the knowledge score was derived from a questionnaire with limited validity evidence, so the absence of an association should not be taken as proof that knowledge is irrelevant. Rather, within this sample, higher knowledge assessment scores were not accompanied by lower stigma, raising the possibility that information provision alone may be insufficient to reduce stigmatizing attitudes. This result supports meta-analyses showing that stigmatization relates to emotional and behavioral factors such as fear, anxiety, lack of empathy, and attitudes rather than cognitive factors (Pettigrew & Tropp, 2008). It has been reported that HIV stigmatization continues despite mandatory continuing medical education (Houston et al., 2019) and that HIV/AIDS education alone is inadequate in reducing stigmatization (Varas-Díaz & Marzán-Rodríguez, 2007). These observations generate a hypothesis rather than demonstrate a fact. HIV-related education programs confined to information transfer may be insufficient on their own. Knowledge may provide the basis for informed attitudes without in itself changing them (Stutterheim et al., 2014). Approaches that additionally target attitudes and behavior may therefore be required, and such approaches have been argued to need to operate at multiple levels (Mahajan et al., 2008). This possibility can only be evaluated in interventional studies. Employment in an internal medicine department or at a university hospital was associated with higher knowledge assessment scores. Previous experience of caring for PLHIV was also associated with higher scores. Because the design is cross-sectional, these associations cannot establish that structured education or clinical contact increases factual knowledge. The direction of the relationship cannot be determined here. Staff with greater interest in the topic may also seek out such settings. No corresponding pattern was observed for stigmatization. Hospital of employment was not significantly associated with any of the three stigma subdimensions (Table 4). The knowledge advantage in these groups was therefore not matched by lower stigma, which is consistent with the hypothesis outlined above.

Some stigmatizing attitudes stand out in our study. First, blaming PLHIV for infection was a widespread view. A significant portion of participants believed that HIV infection is mostly acquired through risky behaviors and thought that individuals would not become infected with HIV if they displayed responsible behavior. This blaming attitude is consistent with findings from Dutch healthcare settings and reflects a broader societal tendency to associate HIV/AIDS with behaviors and identities that are subject to moral disapproval, including drug use and same-sex relationships (Stutterheim et al., 2014). This attitude may negatively affect PLHIV’s access to healthcare services and make the treatment process difficult.

When knowledge level about HIV transmission routes was evaluated, the majority of participants had accurate knowledge about transmission through childbirth and breastfeeding. Similar to our study, there is high awareness in the literature about the protective effect of condom use. However, false beliefs that it can be transmitted through mosquito bites, use of public toilets, and sharing common items show that these topics need clarification in in-service training. Because “I don’t know” responses were scored as incorrect, part of the observed knowledge gap reflects uncertainty rather than active misconception; these items nonetheless indicate the specific topics where educational reinforcement is most needed.

Regarding the item asking whether AIDS is a treatable disease, only about half of participants responded affirmatively. This proportion is comparable to previous reports (Badahdah, 2010). The item is worded in general terms. It does not directly establish whether participants are aware that HIV infection can be durably controlled with ART; the two are related but not equivalent. The low proportion of affirmative responses nonetheless suggests that current treatment advances are not being communicated effectively.

### Study Limitations

This study has several limitations. First, the cross-sectional design precludes causal inference; the reported associations—such as that between seniority and stigma—describe co-occurrence rather than causation. Second, the study was restricted to physicians and nurses in a single city, and the overall response rate was low (21.7%). Because the survey was anonymous, non-responders could not be characterised or compared with responders, so self-selection (non-response) bias cannot be excluded and the sample may not be fully representative of the region’s healthcare workers.

The likely direction of these biases is worth noting. Given the sensitive nature of the topic, social desirability bias would most plausibly lead to under-reporting of stigmatizing attitudes, implying that the true level of stigma may be higher than observed. Conversely, individuals with stronger views about—or greater interest in—HIV may have been more inclined to respond, which could bias estimates in either direction.

Several further limitations concern measurement. The data were collected in 2024 and reflect attitudes at that time; healthcare workers’ knowledge and attitudes may shift with evolving epidemiology, curricula, and clinical exposure. In addition, the knowledge questionnaire was adapted from a previously used instrument but did not undergo formal content-validity quantification (CVI/CVR) or pilot testing in this sample, so the knowledge scores should be interpreted with caution. The questionnaire also did not address misconceptions related to sexual orientation or gender identity, including stigma toward LGBTQIA+ populations—an important dimension of HIV-related stigma that warrants dedicated examination in future studies.

A further measurement limitation concerns what the HPASS captures. The scale assesses self-reported attitudes and behavioral intentions rather than discriminatory behavior observed in clinical practice. The present findings therefore describe what participants report they would think or do, not what they actually do. Establishing whether these reported intentions translate into behavior would require observational or simulated-patient designs.

Furthermore, care experience with PLHIV was assessed as a binary variable (yes/no), without capturing the frequency or nature of such encounters. The heterogeneity of care experiences among participants—ranging from a single incidental contact to regular clinical follow-up—could not be accounted for in the analyses, which may have limited our ability to detect more nuanced associations between care experience and stigmatization levels. Future studies should therefore capture the frequency, nature (e.g., direct clinical contact versus administrative involvement), and duration of care experiences to clarify these associations.

Integrating observational and qualitative approaches could provide a deeper understanding of these findings. The inclusion of staff from three institutions representing different tiers of the public healthcare system broadened the range of professional profiles captured. Given the convenience sampling and the low response rate noted above, however, this breadth should not be taken as evidence that the sample is representative.

## 5. Conclusions

HIV-related stigma among healthcare workers remains a global concern. In this cross-sectional sample, stigmatizing attitudes were common and were associated with sociodemographic and occupational factors—most notably seniority, indexed by age and professional experience—rather than with knowledge assessment scores. Because higher knowledge assessment scores were not accompanied by lower stigma, these findings generate a hypothesis rather than a conclusion: information provision by itself may be insufficient to reduce stigma. The design was cross-sectional, no educational intervention was evaluated, and the knowledge questionnaire carried limited validity evidence in this sample.

These results point to the potential value of interventions that go beyond factual education to address the emotional, moral, and professional-culture dimensions of stigma, for example through case-based and contact-based approaches, promotion of professional ethics, consistent application of universal precautions, and institutional support. Given the associational nature of the evidence, longitudinal and interventional studies are needed to confirm these relationships and to evaluate which strategies most effectively reduce stigma and improve equitable care for PLHIV.

In particular, the high proportion of participants who reported feeling the need to inform other healthcare professionals when encountering a PLHIV may raise confidentiality concerns and points to the value of explicit training in patient confidentiality and non-judgmental, respectful communication.

## Figures and Tables

**Table 1:** Sociodemographic and Occupational Characteristics of the Study Group.

Variable	Total (*n* = 460)	Nurse (*n* = 224)	Faculty Member (*n* = 24)	Specialist Physician (*n* = 92)	Research Assistant (*n* = 120)
**Age (years)**					
Median (IQR)	35.0 (31.0–44.0)	38.5 (32.0–45.0)	54.0 (49.8–57.2)	37.0 (32.8–46.2)	30.0 (27.8–35.0)
**Gender**					
Female	292 (63.5%)	179 (79.9%)	16 (66.7%)	38 (41.3%)	59 (49.2%)
Male	168 (36.5%)	45 (20.1%)	8 (33.3%)	54 (58.7%)	61 (50.8%)
**Marital Status**					
Married	325 (70.7%)	157 (70.1%)	20 (83.3%)	73 (79.3%)	75 (62.5%)
Single	135 (29.3%)	67 (29.9%)	4 (16.7%)	19 (20.7%)	45 (37.5%)
**Hospital of Employment**					
University Hospital	200 (43.5%)	80 (35.7%)	23 (95.8%)	9 (9.8%)	88 (73.3%)
City Hospital	158 (34.3%)	91 (40.6%)	1 (4.2%)	38 (41.3%)	28 (23.3%)
State Hospital	102 (22.2%)	53 (23.7%)	0 (0.0%)	45 (48.9%)	4 (3.3%)
**Specialty**					
Internal Medicine	256 (55.7%)	128 (57.1%)	16 (66.7%)	35 (38.0%)	77 (64.2%)
Surgical	204 (44.3%)	96 (42.9%)	8 (33.3%)	57 (62.0%)	43 (35.8%)
**Professional Experience (years)**					
Median (IQR)	12.0 (6.0–22.0)	16.0 (10.0–25.0)	30.0 (24.8–35.0)	12.0 (8.0–20.0)	4.0 (3.0–8.0)
**Providing care for PLHIV**					
Yes	287 (62.4%)	137 (61.2%)	10 (41.7%)	65 (70.7%)	75 (62.5%)
No	173 (37.6%)	87 (38.8%)	14 (58.3%)	27 (29.3%)	45 (37.5%)
**Self-assessed HIV/AIDS knowledge level**					
Sufficient	121 (26.3%)	62 (27.7%)	6 (25.0%)	23 (25.0%)	30 (25.0%)
Moderate	285 (62.0%)	137 (61.2%)	15 (62.5%)	59 (64.1%)	74 (61.7%)
Insufficient	54 (11.7%)	25 (11.2%)	3 (12.5%)	10 (10.9%)	16 (13.3%)

Data are presented as median (interquartile range, IQR) for continuous variables and *n* (%) for categorical variables. Occupational categories were defined as follows: nurse—registered nurses providing direct patient care; research assistant—physicians undertaking specialty training (residents); specialist physician—physicians who have completed specialty training; faculty member—academic physicians holding university appointments with teaching and research responsibilities. Perceived knowledge level: single-item self-assessment rated by the participant; knowledge assessment score: total score calculated from the 10-question knowledge assessment (range 0–10). Bold text indicates variable and category headings and is used for structural clarity only; it does not denote statistical significance.

**Table 2:** Participants’ Responses to HIV/AIDS Knowledge Questions.

Questions	Answers	*n* (%)
Do you think AIDS is a treatable disease?	Yes	240 (52.2%)
	No	181 (39.3%)
	I don’t know	39 (8.5%)
Do you think HIV transmission can be prevented with treatment?	Yes	283 (61.5%)
	No	115 (25.0%)
	I don’t know	62 (13.5%)
Do you think HIV infection can be transmitted during childbirth?	Yes	415 (90.2%)
	No	33 (7.2%)
	I don’t know	12 (2.6%)
Do you think HIV infection can be transmitted through breastfeeding?	Yes	302 (65.7%)
	No	115 (25.0%)
	I don’t know	43 (9.3%)
Do you think HIV infection can be transmitted by mosquitoes?	Yes	93 (20.2%)
	No	272 (59.1%)
	I don’t know	95 (20.7%)
Do you think HIV infection can be transmitted through daily contact like sharing a public toilet?	Yes	111 (24.1%)
	No	315 (68.5%)
	I don’t know	34 (7.4%)
Do you think HIV infection can be transmitted by coughing, sneezing, or shaking hands?	Yes	48 (10.4%)
	No	388 (84.3%)
	I don’t know	24 (5.2%)
Do you think HIV infection can be transmitted by sharing forks, spoons, or towels?	Yes	123 (26.7%)
	No	293 (63.7%)
	I don’t know	44 (9.6%)
Do you think condom use can prevent HIV transmission?	Yes	372 (80.9%)
	No	64 (13.9%)
	I don’t know	24 (5.2%)
Do you think people with a sexually transmitted disease are more likely to get HIV?	Yes	366 (79.6%)
	No	49 (10.7%)
	I don’t know	45 (9.8%)

Data are presented as *n* (%).

**Table 3:** Distribution of Responses to Health Care Provider HIV/AIDS Stigma Scale (HPASS) Items.

Scale Items	Median	IQR	% Agree (4–5)
**Stereotyping Sub-dimension (14 items)**			
I think PLHIV would not have contracted it if they had sex with fewer people.	3.0	2.0–4.0	46.1%
I avoid performing certain procedures on PLHIV.	2.0	2.0–4.0	26.7%
PLHIV tend to have many sexual partners.	3.0	3.0–4.0	46.5%
I prefer not to have physical contact with PLHIV.	3.0	2.0–4.0	40.9%
I think many PLHIV probably have substance abuse problems.	3.0	3.0–4.0	48.9%
I would rather see an HIV-negative patient than PLHIV.	4.0	2.0–4.0	50.2%
PLHIV should accept responsibility for getting the virus.	4.0	3.0–5.0	63.3%
PLHIV make me uncomfortable.	3.0	2.0–4.0	34.6%
I hesitate to order blood tests for PLHIV because I am concerned for the safety of other healthcare workers.	2.0	2.0–3.0	21.3%
The thought of touching PLHIV is a bit scary to me.	2.0	2.0–4.0	32.0%
It would make me uncomfortable to know that one of my colleagues is person living with HIV.	3.0	2.0–4.0	35.4%
PLHIV through intravenous drug use have acted more irresponsibly than patients infected through blood transfusion.	4.0	4.0–5.0	75.4%
PLHIV through sex have acted more irresponsibly in getting HIV than patients infected through a blood transfusion.	4.0	3.0–5.0	70.2%
It would be difficult to react calmly if a patient told me they were PLHIV.	2.0	1.0–3.0	13.7%
**Discrimination Sub-dimension (12 items)**			
I believe I have the right to refuse treatment to PLHIV for the safety of other patients.	2.0	2.0–3.0	24.8%
PLHIV threaten my health.	3.0	2.0–4.0	35.0%
PLHIV pose a danger to the health of other patients.	3.0	2.0–4.0	34.3%
If I encounter someone who is PLHIV, I feel the need to inform other healthcare professionals.	4.0	4.0–5.0	81.1%
I believe I have the right to refuse to treat PLHIV if I feel uncomfortable.	2.0	2.0–4.0	25.2%
I would want to wear double gloves when examining PLHIV.	3.0	2.0–4.0	39.8%
I believe I have the right to refuse treatment to PLHIV to protect myself.	2.0	2.0–4.0	25.7%
I can work comfortably with a healthcare professional who has HIV. ^a^	4.0	3.0–5.0	69.1%
I believe I have the right to refuse to treat PLHIV if I have concerns about legal liability.	2.0	2.0–3.0	24.8%
I am concerned about contracting HIV from PLHIV.	4.0	2.0–4.0	54.6%
I am concerned that universal precautions are not good enough to protect me from PLHIV.	3.0	2.0–4.0	41.5%
I feel that PLHIV do not share the same values as me.	3.0	2.0–3.0	24.3%
**Prejudice Sub-dimension (4 items)**			
I believe that most PLHIV get the virus through risky behaviors.	4.0	3.0–4.0	67.4%
I think that PLHIV engage in risky activities despite knowing the risks associated with the virus.	4.0	3.0–4.0	58.7%
I think that people will not get HIV if they engage in responsible behaviors.	4.0	4.0–5.0	75.0%
I often think that the health problems of PLHIV are the result of their own irresponsible behavior.	3.0	2.0–4.0	41.1%

Data are presented as median (interquartile range, IQR) for each item. Responses were measured on a 5-point Likert scale where 1 = Strongly Disagree, 2 = Disagree, 3 = Neutral, 4 = Agree, 5 = Strongly Agree. ^a^ Reverse-coded item. Raw (uncoded) responses are presented in this table for interpretability; the item was reverse-coded before computing the discrimination subdimension score and before calculating item endorsement percentages, so that higher subdimension scores consistently indicate higher stigmatization. Bold text indicates subdimension headings only and does not denote statistical significance.

**Table 4:** Effect of Sociodemographic and Occupational Factors on Knowledge and Stigmatization Levels.

	Knowledge Assessment Score	*p*	Stereotyping Level	*p*	Discrimination Level	*p*	Prejudice Level	*p*
**Parameters**								
**Gender:**								
Female	7.0 (6.0–9.0)	0.114	44.0 (37.0–50.0)	0.914	36.0 (29.0–42.0)	0.099	14.0 (12.0–16.0)	0.466
Male	7.0 (6.0–9.0)		43.0 (37.8–49.2)		34.0 (28.0–39.0)		15.0 (13.0–16.0)	
**Marital Status:**								
Single	7.0 (6.0–9.0)	0.274	39.0 (34.0–45.5)	<0.001	30.0 (25.5–38.0)	<0.001	14.0 (12.0–16.0)	0.014
Married	7.0 (6.0–9.0)		45.0 (38.0–50.0)		36.0 (30.0–42.0)		15.0 (13.0–16.0)	
**Hospital:**								
University Hospital	8.0(6.0–9.0)	<0.001	42.0 (36.0–50.0)	0.133	34.0 (27.0–39.0)	0.102	15.0 (12.0–16.0)	0.729
City Hospital	7.0 (6.0–8.0)		44.0 (38.0–49.0)		36.0 (29.0–41.0)		14.0 (12.0–16.0)	
State Hospital	7.0 (5.0–8.0)		44.0 (38.0–50.0)		36.0 (29.0–43.0)		14.0 (13.0–16.0)	
**Department:**								
Internal Medicine	7.0 (6.0–9.0)	0.012	43.0 (37.0–49.0)	0.533	35.0 (29.0–41.0)	0.422	14.0 (12.0–16.0)	0.211
Surgical	7.0 (6.0–8.0)		43.0 (38.0–50.0)		34.0 (28.0–41.0)		15.0 (13.0–16.0)	
**Self-Assessment:**								
Sufficient	8.0 (6.0–9.0)	0.003	45.0 (38.0–51.0)	0.019	35.0 (30.0–43.0)	0.019	15.0 (13.0–16.0)	0.307
Moderate	7.0 (6.0–8.0)		43.0 (38.0–49.0)		35.0 (29.0–41.0)		14.0 (12.0–16.0)	
Insufficient	7.0 (5.0–8.0)		39.0 (33.0–48.0)		32.0 (26.0–37.0)		14.0 (12.0–16.0)	
**Care Provision:**								
Provided Care	7.0 (6.0–9.0)	<0.001	43.0 (37.0–49.0)	0.374	34.0 (28.0–41.0)	0.289	14.0 (13.0–16.0)	0.726
Did Not Provide Care	6.0 (5.0–8.0)		44.0 (37.0–50.0)		36.0 (28.0–42.0)		14.0 (12.0–16.0)	
**Profession:**								
Nurse	6.0 (5.0–7.0)	<0.001	45.0 (39.0–50.0)	<0.001	37.0 (30.0–43.0)	<0.001	14.0 (13.0–16.0)	0.343
Physician	8.0 (7.0–9.0)		41.0 (35.0–47.0)		32.0 (26.0–39.0)		14.0 (12.0–16.0)	
**Physician (Title):**								
Faculty Member	9.0(7.0–10.0)	0.277	55.0 (47.0–58.0)	<0.001	45.0 (39.0–50.0)	<0.001	15.0 (12.0–17.0)	0.382
Specialist Physician	8.0 (6.0–9.0)		42.0 (38.0–49.0)		35.0 (31.0–41.0)		15.0 (13.0–16.0)	
Research Assistant	8.0 (7.0–9.0)		38.0 (31.0–43.0)		28.0 (24.0–33.0)		14.0 (12.0–16.0)	
**Physician (Institution):**								
University Hospital	8.0(7.0–9.0)	0.032	40.0 (33.0–47.0)	0.345	32.0 (26.0–39.0)	0.479	14.0 (12.0–16.0)	0.807
City Hospital	8.0 (7.0–9.0)		41.0 (37.0–46.0)		32.0 (28.0–38.0)		14.0 (12.0–16.0)	
State Hospital	7.0 (6.0–9.0)		41.0 (37.0–50.0)		34.0 (27.0–40.0)		15.0 (13.0–16.0)	

Data are presented as median (interquartile range, IQR). Statistical comparisons were performed using the Mann–Whitney U test for two groups and the Kruskal–Wallis test for three or more groups. *p* < 0.05 was considered statistically significant. Perceived knowledge level: single-item self-assessment rated by the participant; Knowledge assessment score: total score calculated from the 10-question knowledge assessment (range 0–10). Subdimension scores represent the sum of individual item scores (each rated 1–5) within each subdimension. Stereotyping: 14 items (possible range 14–70); Discrimination: 12 items (possible range 12–60); Prejudice: 4 items (possible range 4–20). Higher scores indicate higher levels of stigmatization. Bold text indicates variable and category headings and is used for structural clarity only; it does not denote statistical significance.

**Table 5:** Correlation Analysis Results Among Variables.

		Knowledge Assessment Score	Stereotyping Level	Discrimination Level	Prejudice Level
**Age**	r	−0.002	0.767 **	0.758 **	0.272 **
	*p*	0.960	<0.001	<0.001	<0.001
**Professional Experience**	r	−0.071	0.806 **	0.787 **	0.271 **
	*p*	0.130	<0.001	<0.001	<0.001
**Knowledge Assessment Score**	r	-	−0.045	−0.030	−0.063
	*p*	-	0.332	0.515	0.181

Correlation coefficients (r) were calculated using Spearman’s rank correlation test. ** *p* < 0.01. Age: participant’s age in years; professional experience: duration of professional experience in years; knowledge assessment score: total score from the 10-question knowledge assessment (range 0–10); Stereotyping, Discrimination, Prejudice: subdimension scores of the Health Care Provider HIV/AIDS Stigma Scale (HPASS). Spearman’s rank correlation coefficient (rs) was used. *p* < 0.05 was considered statistically significant. Bold text indicates variable and category headings and is used for structural clarity only; it does not denote statistical significance.

**Table 6:** Multivariable linear regression models for stereotyping, discrimination, and prejudice scores.

	Stereotyping Level		Discrimination Level		Prejudice Level	
Predictor	*B* (95% CI)	*p*	*B* (95% CI)	*p*	*B* (95% CI)	*p*
***Model 1 (including age)***						
**Age (per year)**	0.74 (0.67 to 0.82)	0.001	0.70 (0.64 to 0.76)	0.001	0.11 (0.07 to 0.14)	0.001
Female	−0.72 (−2.09 to 0.71)	0.287	0.28 (−0.91 to 1.52)	0.653	−0.12 (−0.64 to 0.40)	0.677
Married	1.16 (−0.04 to 2.34)	0.048	0.99 (−0.18 to 2.12)	0.110	0.35 (−0.21 to 0.90)	0.263
Faculty member	−2.65 (−5.62 to 0.12)	0.076	−2.25 (−4.80 to 0.38)	0.106	−1.50 (−3.30 to 0.06)	0.058
Specialist physician	−2.30 (−4.10 to −0.48)	0.008	−1.95 (−3.66 to −0.35)	0.030	−0.02 (−0.85 to 0.72)	0.953
Research assistant	−2.76 (−4.40 to −0.99)	0.004	−3.48 (−5.15 to −1.99)	0.001	0.25 (−0.61 to 1.09)	0.536
City hospital	−0.08 (−1.54 to 1.27)	0.908	−0.57 (−1.97 to 0.78)	0.441	−0.39 (−1.05 to 0.27)	0.245
State hospital	−0.40 (−2.27 to 1.40)	0.640	−0.47 (−2.21 to 1.22)	0.625	−0.40 (−1.21 to 0.40)	0.340
Surgical department	0.45 (−0.62 to 1.64)	0.424	−0.64 (−1.80 to 0.53)	0.292	0.33 (−0.18 to 0.77)	0.234
Care experience with PLHIV: yes	−0.19 (−1.44 to 1.19)	0.797	−0.29 (−1.56 to 0.99)	0.654	−0.22 (−0.87 to 0.40)	0.429
Perceived knowledge: moderate	−0.86 (−2.26 to 0.74)	0.242	−0.62 (−2.13 to 0.85)	0.400	−0.20 (−0.84 to 0.47)	0.536
Perceived knowledge: insufficient	−1.70 (−3.96 to 0.65)	0.141	−1.78 (−3.85 to 0.27)	0.068	−0.47 (−1.47 to 0.50)	0.357
Knowledge assessment score (per point)	−0.10 (−0.41 to 0.25)	0.564	0.01 (−0.31 to 0.37)	0.958	−0.13 (−0.29 to 0.02)	0.089
*Model R^2^ (adjusted R^2^)*	*0.596 (0.584)*		*0.597 (0.585)*		*0.115 (0.089)*	
***Model 2 (including work experience)***						
**Work experience (per year)**	0.74 (0.68 to 0.81)	0.001	0.68 (0.62 to 0.74)	0.001	0.10 (0.07 to 0.13)	0.001
Female	−1.32 (−2.56 to −0.04)	0.040	−0.29 (−1.45 to 0.88)	0.643	−0.21 (−0.77 to 0.37)	0.444
Married	1.10 (−0.10 to 2.31)	0.065	1.01 (−0.39 to 2.30)	0.127	0.36 (−0.19 to 0.94)	0.233
Faculty member	−1.28 (−4.43 to 1.76)	0.384	−0.74 (−3.87 to 2.28)	0.605	−1.27 (−2.82 to 0.19)	0.094
Specialist physician	0.12 (−1.56 to 1.74)	0.890	0.27 (−1.48 to 2.14)	0.742	0.32 (−0.39 to 1.03)	0.395
Research assistant	−0.56 (−2.19 to 1.16)	0.512	−1.60 (−3.15 to 0.00)	0.063	0.53 (−0.33 to 1.42)	0.224
City hospital	−0.72 (−2.06 to 0.72)	0.298	−1.14 (−2.55 to 0.26)	0.089	−0.48 (−1.12 to 0.15)	0.129
State hospital	−0.84 (−2.54 to 0.79)	0.307	−0.84 (−2.63 to 1.14)	0.342	−0.46 (−1.28 to 0.30)	0.269
Surgical department	0.48 (−0.60 to 1.47)	0.397	−0.61 (−1.67 to 0.38)	0.265	0.33 (−0.19 to 0.83)	0.203
Care experience with PLHIV: yes	−0.22 (−1.47 to 0.97)	0.716	−0.33 (−1.62 to 1.01)	0.619	−0.23 (−0.80 to 0.32)	0.430
Perceived knowledge: moderate	−0.46 (−1.81 to 0.99)	0.480	−0.28 (−1.75 to 1.19)	0.697	−0.15 (−0.72 to 0.46)	0.620
Perceived knowledge: insufficient	−1.45 (−3.59 to 0.62)	0.187	−1.63 (−3.52 to 0.38)	0.107	−0.44 (−1.44 to 0.55)	0.357
Knowledge assessment score (per point)	−0.06 (−0.35 to 0.24)	0.723	0.05 (−0.28 to 0.40)	0.751	−0.13 (−0.27 to 0.03)	0.097
*Model R^2^ (adjusted R^2^)*	*0.630 (0.619)*		*0.609 (0.597)*		*0.117 (0.091)*	

*B*: unstandardized regression coefficient; CI: confidence interval. Confidence intervals and *p* values were derived from bias-corrected and accelerated (BCa) bootstrapping with 1000 resamples; the smallest *p* value obtainable with this number of resamples is 0.001. Because age and work experience were highly collinear (Spearman’s rs = 0.946; variance inflation factor > 16 when entered together), they were modelled separately: Model 1 includes age and Model 2 includes work experience, with all other covariates identical. In both models the maximum variance inflation factor was below 2.0. Reference categories: male (sex), single (marital status), nurse (profession), university hospital (institution), internal medicine (department), no previous care experience with PLHIV, and sufficient perceived knowledge. Knowledge assessment score: total score from the 10-item knowledge questionnaire (range 0–10). Higher subdimension scores indicate higher stigmatization. For the married-versus-single contrast in Model 1 (stereotyping), the BCa confidence interval marginally includes zero while the corresponding bootstrap *p* value falls just below 0.05. Because the bias-corrected and accelerated interval and the bootstrap *p* value are derived by different procedures, and because this estimate lies very close to the conventional threshold, the two can diverge; resampling variability alone can move the *p* value across 0.05 for an estimate of this magnitude. This association is therefore regarded as borderline and not robust, consistent with Model 2, in which marital status was not significantly associated with stereotyping (*p* = 0.065). Analyses were performed in IBM SPSS Statistics v26. Bold text indicates model and predictor headings; italics are used only for statistical symbols (*B*, *p*, *n*). Neither denotes statistical significance.

## Data Availability

The datasets used and/or analyzed during the current study are available from the corresponding author on reasonable request. The data are not publicly available due to privacy and ethical restrictions.

## Supplementary Materials

The following supporting information can be downloaded at: https://www.mdpi.com/article/10.3390/bs16091681/s1. Table S1: STROBE Statement—checklist of items that should be included in reports of cross-sectional studies; Table S2: Effect sizes for group comparisons of knowledge assessment score and stigmatization scores.

## Abbreviations

AIDS	Acquired Immunodeficiency Syndrome
ART	Antiretroviral Therapy
BCa	Bias-Corrected and Accelerated (bootstrap)
CI	Confidence Interval
HIV	Human Immunodeficiency Virus
HPASS	Health Care Provider HIV/AIDS Stigma Scale
IQR	Interquartile Range
PLHIV	People Living with HIV
SPSS	Statistical Package for the Social Sciences
UNAIDS	Joint United Nations Programme on HIV and AIDS
WHO	World Health Organization
