# Supplementary material for: Knowledge and HIV-Related Stigmatizing Attitudes Among Healthcare Workers: A Cross-Sectional Study
Source: Behav Sci (Basel). 2026 Sep 18;16(9):1681. doi: 10.3390/bs16091681 (PMC13603121; doi:10.3390/bs16091681)
Supplement: Supplementary file 1 [file behavsci-16-01681-s001.zip › behavsci-4425607-supplementary.pdf]

Table S1. STROBE Statement—checklist of items that should be included in reports of cross-sectional studies.

Section references correspond to the revised manuscript. Page numbers are to be completed at the final typesetting stage.

Item	Recommendation	Reported in (section) / Page
Title and Abstract		
1 (a)	Indicate the study's design with a commonly used term in the title or abstract	Title; Abstract
1 (b)	Provide in the abstract an informative and balanced summary of what was done and what was found	Abstract
Introduction		
2	Explain the scientific background and rationale for the investigation	Introduction
3	State specific objectives, including any prespecified hypotheses	Introduction (final paragraph); Abstract
Methods		
4	Present key elements of the study design early in the paper	Section 2.1 (Study design)
5	Describe the setting, locations, and relevant dates, including the data-collection period	Section 2.2 (Setting); dates 15 Aug–30 Sep 2024
6 (a)	Give the eligibility criteria and the sources and methods of selection of participants	Section 2.3 (Participants and sampling)
7	Clearly define all outcomes, exposures, predictors, potential confounders, and effect modifiers	Section 2.5 (Measures); Section 2.6
8	For each variable of interest, give sources of data and details of methods of assessment (measurement)	Sections 2.4 and 2.5
9	Describe any efforts to address potential sources of bias	Section 2.3 (self-selection acknowledged); Discussion (Limitations)
10	Explain how the study size was arrived at	Section 2.3 (Cochran's formula with finite population correction)
11	Explain how quantitative variables were handled; describe groupings and why	Sections 2.5 and 2.6
12 (a)	Describe all statistical methods, including those used to control for confounding	Section 2.6 (Statistical analysis)
12 (b)	Describe any methods used to examine subgroups and interactions	Section 2.6 (multivariable models); Results
12 (c)	Explain how missing data were addressed	Section 2.3 (complete-case analysis; only fully completed forms retained)
12 (d)	If applicable, describe analytical methods taking account of sampling strategy	Not applicable (total-population invitation)
12 (e)	Describe any sensitivity analyses	Section 2.6 (separate models for collinear predictors: age vs. work experience)
Results		
13 (a)	Report the numbers of individuals at each stage of the study	Section 3 (Results); 2,230 invited, 483 questionnaires submitted, 23 excluded for incomplete data, 460 analyzed
13 (b)	Give reasons for non-participation at each stage	Results (response rate; non-responders not characterisable)

Item	Recommendation	Reported in (section) / Page
13 (c)	Consider use of a flow diagram	Not applicable
14 (a)	Give characteristics of study participants and information on exposures and potential confounders	Results, Table 1
14 (b)	Indicate the number of participants with missing data for each variable	Section 2.3; Results (complete cases; no missing data in analysed sample)
15	Report numbers of outcome events or summary measures	Results, Tables 2–4
16 (a)	Give unadjusted and, if applicable, confounder-adjusted estimates with their precision	Results, Table 4 (unadjusted); Table 6 (adjusted); Table S2 (effect sizes)
16 (b)	Report category boundaries when continuous variables were categorized	Section 2.5; Results/Tables (score ranges)
16 (c)	If relevant, translate estimates of relative risk into absolute risk	Not applicable
17	Report other analyses done (e.g., subgroups, sensitivity analyses)	Results, Table 6; Table S2
Discussion		
18	Summarise key results with reference to study objectives	Discussion (opening paragraph)
19	Discuss limitations, including the direction and magnitude of any potential bias	Discussion (Limitations)
20	Give a cautious overall interpretation considering objectives, limitations, and other evidence	Discussion
21	Discuss the generalisability (external validity) of the results	Discussion (Limitations / generalisability)
Other Information		
22	Give the source of funding and the role of the funders	Funding statement

An Explanation and Elaboration article discusses each checklist item and is available at www.strobe-statement.org.

Table S2. Effect sizes for group comparisons of knowledge assessment score and stigmatization scores.

Comparison	Knowledge	Stereotyping	Discrimination	Prejudice
Mann–Whitney U test — rank-biserial r				
Gender (female vs. male)	0.09	0.01	0.09	0.04
Marital status (married vs. single)	0.06	**0.30**	**0.28**	**0.15**
Department (internal vs. surgical)	**0.13**	0.03	0.04	0.07
Care experience (yes vs. no)	**0.27**	0.05	0.06	0.02
Profession (physician vs. nurse)	**0.49**	**0.25**	**0.27**	0.05
Kruskal–Wallis test — epsilon-squared (ε^2)				
Hospital (3 groups)	**0.04**	0.01	0.01	<0.01
Self-assessed knowledge (3 groups)	**0.03**	**0.02**	**0.02**	0.01
Physician title (3 groups)	0.01	**0.24**	**0.29**	0.01
Physician institution (3 groups)	**0.03**	0.01	0.01	<0.01

Effect sizes correspond to the group comparisons reported in Table 4. Rank-biserial correlation (r) is reported for two-group Mann–Whitney U tests and epsilon-squared (ε^2) for Kruskal–Wallis tests with three groups. For r, values of 0.10, 0.30, and 0.50 indicate small, medium, and large effects; for ε^2, values of 0.01, 0.06, and 0.14 indicate small, medium, and large effects, respectively.

Bold values denote comparisons that were statistically significant ($p < 0.05$) in Table 4. Physician title and physician institution comparisons were restricted to physicians (n = 236). Knowledge: total score from the 10-item questionnaire (range 0–10); Stereotyping, Discrimination, and Prejudice: HPASS subdimension scores.
